# Replicating or franchising a STEM afterschool program model: core elements of programmatic integrity

**DOI:** 10.1186/s40594-021-00320-0

**Published:** 2022-01-28

**Authors:** Nikolaus Stevenson, Amie S. Sommers, Neal Grandgenett, William Tapprich, Julia McQuillan, Michelle Phillips, Rachael Jensen, Christine Cutucache

**Affiliations:** 1 STEM Teaching, Research, and Inquiry-Based Learning (TRAIL), University of Nebraska at Omaha, Omaha, NE USA; 2 Department of Biology, University of Nebraska at Omaha, Omaha, NE AH114 USA; 3 School of Natural Resources, University of Nebraska-Lincoln, Lincoln, NE USA; 4 Department of Teacher Education, University of Nebraska at Omaha, Omaha, NE USA; 5 Department of Sociology, University of Nebraska-Lincoln, Lincoln, NE USA; 6 Worlds of Connections SEPA Project, University of Nebraska-Lincoln, Lincoln, NE USA; 7 Phillips and Associates, San Francisco, CA USA; 8 Youth Safety Office, University of Nebraska at Omaha, Omaha, NE USA

**Keywords:** NE STEM 4U, Out-of-school time programming, Afterschool program, Outreach, STEM, Program fidelity, Educational organizational leadership

## Abstract

**Background:**

Designed in 2012 with a first implementation in 2013, NE STEM 4U is a professional development program for post-secondary students/undergraduates, and serves as a source of outreach, content knowledge generation, and STEM literacy for youth in grades kindergarten through 8th grade (ages 5–14). The model empowers post-secondary students as facilitators of inquiry-based learning within the context of an out-of-school time program. This study investigated the potential for replicating or ‘franchising’ this model by evaluating on the following: (1) Is the model replicable? And, if so, (2) what core elements are necessary for program fidelity? And (3) is there a dependency on a particular setting/participant type (e.g., a more rural or urban setting)?

**Results:**

Strategic expansion of the program to different institutional types (i.e., Research 1, Research II, and a predominantly undergraduate institution), different geographical locations (i.e., rural and urban), and with various school district partners (i.e., large and small) determined that program fidelity and replicability required 4 core elements or criteria: (i) intentional programming, (ii) staff quality, (iii) effective partnerships, and (iv) program evaluation and continuous improvement. Importantly, we examined emergent themes by each site, as well as in combination (*n* = 16 focus group participants, *n* = 12 reflection surveys). These data indicated that *Flexibility* (21.22%)*, Student Engagement (i.e., Youth)* (19.53%)*, Classroom Management (i.e., also pertaining to youth)* (19.31%)*, and Communication* (15.71%) were the themes most referenced by the post-secondary student mentors in the NE STEM 4U program, regardless of site. Finally, the YPQA results demonstrate general replication of program quality in a “franchise” location.

**Conclusions:**

These results highlight the core elements of the NE STEM 4U program for consideration of expansion (through strategic replication or ‘franchising’) as a possible international model. The findings and voices highlight the program’s trajectory toward success into environments that expand professional development for post-secondary students, and for delivering STEM opportunities for youth.

**Supplementary Information:**

The online version contains supplementary material available at 10.1186/s40594-021-00320-0.

## Introduction

In the race for talent, all components of the workforce require access to highly skilled students earlier and earlier in their educational pathways (Camilli & Hira, 2019). For example, post-secondary (i.e., undergraduate) students may participate in an internship or other experiential learning opportunity with prospective corporations and non-profit or governmental entities well before graduation. Experiences in applied settings allow employers to identify skilled students who might contribute to their organization and allow students to understand what will be most important for them to learn for later success (Carnevale & Smith, 2013). Many employers seek graduates with science, technology, engineering, and mathematics (STEM) degrees and these graduates routinely obtain jobs with higher salaries than those graduating with degrees in other fields (Noonan, 2017). Given the current employment landscape, it is important for institutions of higher education to retain such students, help them to complete their STEM degrees, and have experiences that add value to the interest of future employers.

Retention in STEM programs is not an easy institutional task; despite an emphasis on retention in higher education, many post-secondary students struggle to complete a degree in STEM fields (American Association for the Advancement of Science, 2011; Dolan, 2017; Eddy & Hogan, 2014; Egenrieder, 2010; Gottesman & Hoskins, 2013). Consequently, the lack of retention to graduation leads to a shortage of STEM professional workers. Many factors contribute to STEM graduates, such as selecting a major aligned with their interests, and persisting through the program. Some of those factors are relatively distal (e.g., some children determine their interest in STEM fields as early as fourth grade; Ing & Nylund-Gibson, 2013). The interest in STEM is pervasive during pre-adolescence. However, if students decide that STEM fields are not for them, it is much more challenging to change their minds (Harrison et al., 2011; Husserl, 1913/1983; Hutchinson-Anderson et al., 2015; Maltese and Tai, 2011; Sithole et al., 2017; UNO Advantage [UNO], 2021). Other factors, such as the cost of higher education, may present challenges to families seeking to support and mentor college students (especially first-generation college students; NAS, 2016a, 2016b). In the institutional setting, barriers to persistence such as “weed out” classes also have impact (Dabney et al., 2012, 2013, 2016; DeWitt et al., 2016; Nicholls et al., 2010). In addition, preparation in STEM fields is highly variable in K-12 education systems, thus some college students are better prepared than others for the rigors of higher education (Thiry et al., 2012; Xia et al., 2015). Students who have little exposure to STEM fields or to higher education prior to arriving at the academy can struggle to feel a sense of belonging and/or to navigate through the bureaucratic challenges and promising opportunities in college (Bangera & Brownell, 2014; Heim & Holt, 2019; Kinner & Lord, 2018; Lopatto et al., 2014; Rainey et al., 2018).

### About the NE STEM 4U program

The Nebraska Science, Technology, Engineering and Mathematics 4U Program (NE STEM 4U) was founded in 2012 as a post-secondary student led, faculty-guided program. The program is designed to dually serve post-secondary student mentors as a pre-professional training program, regardless of major while also providing an afterschool education experience for youth using an inquiry-based learning approach.

#### The institution at the site of origin

In an effort to address challenges to persistence in STEM fields in college, such as early life STEM preferences (e.g., 4th to 8th grade) and post-secondary (i.e., undergraduate) student preparation and retention, we created the program NE STEM 4U (NE indicates Nebraska, a state in the central United States, where the program originated; Cutucache et al., 2016; Leas et al., 2017). The NE STEM 4U program began at a large, metropolitan university in an urban setting that (at the time) offered a limited number of masters and doctoral programs. The university is set in a sprawling city with a population of over 500,000. Since the program started, the initiating institution has experienced tremendous growth and productivity in research that led to a research-intensive (i.e., *R*2) designation. Previous studies documenting the impact of the NE STEM 4U program on youth outcomes showed evidence of gains in preferences for STEM careers among youth in 4th through 8th grade, as well as better preparation and persistence in STEM paths among college students (Leas et al., 2017).

The NE STEM 4U program provided many positive outcomes for the originating institution in 8 years of programming thus far and is well positioned to continue. Based upon the success to date, leaders at campuses in the same university system (i.e., the “*R*1” and a “rural/primarily undergraduate institute, PUI” campus) sought to adopt the NE STEM 4U model (i.e., becoming a “franchise”). The term franchise, although nearly synonymous with replication in this context, also represents that the replicating institutions share in funding the program, provide oversight, use shared lesson plans, and, to some degree, work collaboratively across replicating institutions, albeit at the time of this manuscript all replicating institutions reside in the same state within the United States. However, the concept is similar to what a business franchise might do. The ultimate goal for the initiating/founding institution, as well as the franchise sites, is to develop a strategy, process, and dissemination practice to establish a national network of NE STEM 4U programs.

#### What is unique about the NE STEM 4U program design?

Since many “outreach” programs exist, with many involving post-secondary students as mentors (Aldous Bergerson & Peterson, 2009; Hebets et al., 2020; Tenenbaum et al., 2014) for younger students, it is important to describe how our program is unique, as these are also the attributes that have most contributed to the overall sustainability, and impacts reported for our program.

As mentioned, the program was designed to support the pre-professional development/workforce preparation for post-secondary students, who will be referred to as “mentors”. The program has followed a Constructivist Theoretical Framework (Annells, 1996; Bernstein, 1983; Mills et al., 2006) since inception, and this can most succinctly be captured by a tri-fold model of teaching, mentorship, and research as interventions for the participating mentors. Constructivism is embedded due to the need to provide flexibility of experiences for mentors, and to have the opportunity to study which components of the program (1) lead to the most effective preparation of mentors for the workforce (e.g., to measure “soft” and “hard” skill development), and (2) to provide fidelity of program on a long-term basis, to ensure all mentors, particularly persons traditionally excluded due to ethnicity or race (PEERs, Asai 2020) in higher education can be supported in a range of ways, and (3) so that we could determine if dosage (or frequency or style of interaction played a role in the intervention).

#### Program design: teaching

The tri-fold model of the program’s intervention and participation opportunities for post-secondary students include teaching, research, and mentorship. The *teaching* component is carried out by having the mentors facilitate hands-on, minds-on, culturally relevant, pedagogically sound STEM activities with youth (i.e., in grades K-8 in the U.S., or approximately ages 5 through 14). These activities make complex STEM concepts relatable and engaging. The mentors provide this programming in teams of 2–3, and they travel to the site of our participating schools in the out-of-school time hours (i.e., immediately after the formal school day ends) to host programming for 1 h, two times weekly throughout the year. Even during the COVID-19 pandemic (at the time of writing this manuscript), the program has not paused, it shifted to simply remote instruction. The mentors are given extensive, and consistent training to provide this instruction—namely, child safety training, culturally relevant pedagogical training, preparation with the lesson plans that they will carry out via mentored practicing and then practicing more as a team before delivery, and other timely topics via “STEMinars” to include Youth Voice, Youth Leadership, and modeling of high-impact practices through established assessment training including the David P. Weikart Youth Program Quality Assessment (YPQA) instrument, and Dimensions of Success (PEAR, 2020). Effective teaching includes youth actively engaged as scientists in the activities with guidance from mentors. The results of several longitudinal assessments, formative and summative feedback using teaching observation protocols, and via semi-structured interviews to include dosing measurements all indicated significant gains for mentors thanks to the intervention of teaching (Nelson & Cutucache, 2017; Nelson et al., 2018).

#### Program design: research

The mentors (i.e., post-secondary students, or known in the U.S.A. as undergraduates) in the program can participate in research experiences within the NE STEM 4U program, in addition to teaching. These research studies involve mixed methods approaches and human subjects, similar to the social sciences, thereby providing research experiences for traditional STEM majors in areas that are separate from those that they might experience in a course or other apprenticeship style research experience (i.e., the laboratory bench). Specifically, undergraduates participate in discipline-based education research (so called ‘DBER’), which combines the knowledge of teaching/learning with discipline-specific content, further expanding their preparation to include the cognitive sciences and qualitative work, a complement to their (often) quantitative heavy degree plans.

Moreover, the translation of these quantitative and qualitative skills, and the certifications and training needed to work with human subjects have helped graduates in job-seeking, graduate applications, grant applications, and have supported many mentors to be co-authors on presentations, and publications. The uniqueness of bringing translational research opportunities surrounding education, cognitive, and human subjects research have added another element of uniqueness to this program, when compared with others, and are directly in line with national calls to action (NAP, 2017).

#### Program design: mentoring

Mentors in the program both *gain* mentorship and *give* mentorship, which makes this program highly unique. The mentors gain mentorship from near-peers within the institution, and have direct access to faculty advisor support, to include frequent (at least weekly) meetings, advising, assistance with career planning, and ultimately letter preparation with letters being able to speak not only to say performance in a class, but truly to character development and the candidate’s abilities surrounding critical thinking, problem solving, leadership, collaboration, communication, and professionalism, due to the demonstrated actions of these within the NE STEM 4U program. Finally, the mentors also provide mentorship to the youth in the program, helping to serve as role models for them and helping to support their curiosity in STEM while simultaneously expanding their STEM content knowledge (Leas et al., 2017; Nelson & Cutucache, 2017).

### Theoretical framework for replication or franchise of model

The literature focusing on the conceptualization and design of programs that can be replicated are frequently studied, but the replication and scaling aspects often fall short of the goal (Clark, 2008; Hubbard et al., 2006; McNeil, 2002; Stevens et al., 2018). Furthermore, the emphasis of preserved fidelity within program replication likely leads to the preservation of an organization’s core elements (O’Donnell, 2008; Stevens et al., 2018), but without proper ongoing evaluation on the replication sites, the ‘lethal mutations’ described by Brown (Brown, 1992) or the loss of ‘integrity’ (LeMahieu, 2011; Stevens et al., 2018) can lead to confounding objectives by the initiating and replication sites. In addition, the literature on ‘adapting in practice’, or the so called study of the outcomes from replication/franchise of an initiating site’s program remain rare (Stevens et al., 2018); consequently, we aim to provide herein research to expound upon the literature in direct support of ‘adapting in practice’, similar to that described for the FUSE Studios program, now reaching approximately 200 locations (Stevens et al., 2018). Therefore, we utilized the constraints of program ‘integrity’ to ensure preservation of core elements of the NE STEM 4U program, as well as to give a voice to each partnering site to adapt to local constraints (Clark, 2008; Hubbard et al., 2006; McNeil, 2002; Penuel et al., 2011; Ramey et al., 2018; Stevens et al., 2018). These themes aligned with both the core elements of the NE STEM 4U program, and helped us to determine which elements are core, and which are able to be adapted while preserving the programmatic integrity.

The theoretical basis for this model replication is rooted within Implementation Science (Mittman, 2012; Sales et al., 2006). Implementation Science helps to “guide the: (a) understanding of factors or determinants that may influence implementation, and (b) the selection of implementation strategies (or strategies if multifaceted)” (Mittman, 2012; Sales et al., 2006). Namely, we utilize the normalization process theory (NPT) to describe the program comprehensively to demonstrate inputs, outputs, feedback loops, and orientation of these items within the wider programmatic system (May & Finch, 2009; May et al., 2015). Implementation science was derived as a result of approximately a decade (i.e., 1998–2008) of refining a theorem surrounding “empirical generalizations from analysis of data collected in qualitative studies of healthcare work and organization” (Hegger et al., 2016; May & Finch, 2009; May et al., 2009). Furthermore, “the principles of the theory ensure that constructs did not conflict with one another, that it had explanatory power, and possessed sufficient robustness for formal testing” (May et al., 2009; Murray et al., 2010). This theoretical approach has been further expanded outside of solely the healthcare realm and is now utilized as an aid to support complex processes, including programmatic development, testing, assessment, and ultimately replication of educational programs.

Implementation science often uses a decision-tree model to facilitate the aim of a particular program or intervention. Specifically, we use NPT’s set of tools to understand the processes by which “thinking, enacting, and organizing work are operationalized” (Chambers et al., 2013; Grol et al., 2007; ICEBeRG, 2006; May & Finch, 2009; May et al., 2009, 2015). Whenever an educational program is implemented, some processes become embedded, others are constantly refined, and yet others are not fully realized nor appreciated by the designers. Maintenance of program fidelity is critical at each step. It is essential that all of these components are articulated in a logical framework for successful replication into other environments, and particularly when the sites themselves need to take franchise-like ownership at the local level. Consequently, we have identified the programmatic components using the NPT structure, to include our real-time feedback loops, within this manuscript.

We have designed an approach to strategically combat the substantial challenges facing STEM preparation and student retention through the development of the NE STEM 4U program. This program serves as a professional development program for post-secondary STEM majors. To determine the operational success of this program, we evaluated the programmatic structure under the framework of the NPT.

Finally, as determined by Alvesson and Sköldberg (2000) that the researcher or leader reflexivity enhances perspectives, motivations, and perceptions. Thus, we have included voices from the franchise sites to discuss their perspectives, motivations, and the scale-up process in their own words.

### Research questions

The core questions are (1) is the model replicable? And, if so, (2) what core components are necessary for program fidelity? And, (3) is there a dependency on a particular setting/participant type (be that a more rural or urban setting) for program fidelity?

We used several strategies to identify the core contributions that ensure program fidelity and replication, and particularly within a context of franchise-like co-ownership. First, we interviewed post-secondary mentors at both the original and the franchise NE STEM 4U locations. Second, we gathered data from faculty mentors to provide validation and context for interpreting what was learned from the mentors. Finally, we interviewed replication site leads to situate the work within their unique perspectives, goals, and experiences during the scale-up.

## Methodology

### Qualitative research approach and rationale

Guided by the emphasis on perceptions, perspectives, and reflexivity in our implementation science, normalization process framework, we used qualitative sources of data (i.e., focus groups, interviews, and participant observation) from each study site to gather data. We asked participants to describe their experiences and what they thought impacted the successes or challenges affiliated with franchising (replicating) the NE STEM 4U program at their location. We chose the term ‘replication’ or ‘franchise’ to capture the idea that there are core elements of the program that are essential for fidelity, yet each site had unique reasons to adopt the program, support it with significant ownership, and adapt it to be most relevant to local needs. As mentioned, we feel that this approach loosely parallels commercial franchises that must meet certain criteria and establish a replicated environment to be considered a franchise but also have some options that they can modify to adjust to local markets.

Semi-structured interview guides with open-ended questions provided consistency and flexibility for the focus groups and interviews. The goal was to elicit students’ experiences and feelings to understand their perceptions of the program (Lester, 1999; McNamara, 2009; Moustakas, 1994). The participant observation data came from the ongoing communication between the leaders of each site and the program founder and staff, plus the observations of evaluators at each location. It is possible for different locations to emphasize different criteria for success. For example, one might focus on the professional development and retention of the post-secondary students, another might focus on attracting middle school aged youth to STEM careers, and another on serving the needs of local schools by reinforcing content in state standards, supporting vertical scale-up per NPT (May et al., 2009).

One possible barrier to adopting an existing program (becoming a franchise) is concern that it will not be the same program if the franchise makes some minor changes for the local setting. Importantly, we established nonnegotiable core elements of the program to promote fidelity while highlighting strong communication between the initiation and franchise/expansion sites to successfully scale-up. This includes defining assessment, evaluation, and programming outcomes. Ultimately, the initiation site has served as the approver of any programmatic modifications, as a way to ensure that the initial program was indeed being replicated (rather than an evolution of it) similar to how commercial franchises are operationalized. However, we set out to see if the program was robust enough to tolerate minor adaptations to meet local needs and still preserve the core elements of the initiating. For example, minor changes would include the content of lessons delivered, and leadership structure (i.e., more seasoned leaders versus those new to such a program structure). In short, we sought a relatively broad notion of “fidelity” that could capture the essential components to reach the major goals while also allowing for some degree of localized customization.

### Network of NE STEM 4U contributors via semi-structured interviews and surveys

The NE STEM 4U program engages a diverse group of stakeholders, participants, collaborators and contributors, to include those within local non-profit and school leaders. Consequently, to gain feedback on the effectiveness and challenges of franchising the program, we completed semi-structured interviews and administered surveys to core participants from all three sites. The founding or initiating institution (Research 2 (R2)) is in a large (~ 500,000), urban, metropolitan area and has about 15,000 post-secondary students. The Research I (RI) institution replication site is also in a metropolitan setting, but in a more modest sized city (~ 300,000), has about 25,000 students, and it is designated as research intensive PhD granting institution. Finally, the predominantly teaching focused (PUI), research active institutional partner replication site is located in a smaller town (~ 35,000) in a more rural area.

As approved within our Institutional Review Board approvals for the protection of human subjects aligned with national human subject research protection protocols (# 548-12-EX and 015-17-EX), we asked post-secondary students (i.e., undergraduate participants/mentors) in the program to participate in semi-structured phenomenological focus group interviews that lasted between 30 and 40 min or reflection surveys of similar content in early 2020 from all three replication sites (*n* = 16 focus group participants total, *n* = 12 participants from the founding institution, *n* = 2 participants from the RI replication site, and *n* = 2 participants from the PUI replication site; *n* = 12 survey reflections from the PUI replication site) (Cresswell & Poth, 2016; McNamara, 2009; Saldaña, 2015; Yin, 2018). We conducted these interviews in person for the founding site and the RI replication site, and via zoom for the PUI replication site. Post-secondary mentors who participated in the focus groups and reflection surveys had participated in NE STEM 4U as a mentor for at least two semesters, ensuring they had adequate experience to provide their perspectives on NE STEM 4U. Prior to the start of the interviews and reflection surveys, NE STEM 4U mentor participants were asked for their informed consent to participate that included permission for the interview to be recorded. Students were informed that all personal information would be kept confidential (including the removal of identifiers from transcripts and data collection) and were instructed not to use names or other identifiers in the interviews. To ensure students were able to provide informed feedback, the interviews occurred, while all mentors were either still active in the program or within 1 month of completing the program.

According to the semi-structured phenomenological interview framework, we began the interviews by asking students open-ended, guiding questions, including descriptions and perspectives of their general experience in the program, including experiences with youth participants and faculty mentors Additional file 1: Table S1; (Creswell & Poth, 2016; Lester, 1999; McNamara, 2009; Moustakas, 1994). This allowed the interviewer to follow up on participants’ answers, thus allowing the facilitator to gain further insight and detail into their meaning and intention. The interviewer was not an active member of the NE STEM 4U oversight team, nor a mentor, thus allowing students to speak openly and confidentially regarding their experiences while preventing participant and interviewer bias (Creswell & Poth, 2016; McNamara, 2009). The interviewer followed recommended practices for phenomenological interviews (i.e.Creswell, 2007; McNamara, 2009; Smith et al., 2009). These practices were aligned with Moustakas (1994), allowing collection of “rich, vital, substantive descriptions of a phenomenon” and allowed for flexibility with the open-ended responses. Interviews and focus groups for all sites were conducted in person, and surveys were collected electronically. All interviews were subsequently transcribed following recording for accuracy. Several authors coded for themes. In addition, authors that have been involved with originating and franchising NE STEM 4U provided insights from participant observation and regular recorded meetings.

### Analysis of interviews and process to determine emergent themes

To identify emergent themes within the data, we analyzed the transcribed interviews of the participants across all three sites both individually (i.e., “*N*1”, “*N*2”, and “*N*3”), and combined (i.e., “*N*1 + *N*2 + *N*3”) to inform the study (Sanders, 1982). The transcribed interviews (*n* = 16) and reflection surveys (*n* = 12) were coded to include positive and negative nodes to identify preliminary emergent themes. After identification of emergent themes, we examined the coverage of these coded nodes using NVivo 12 (©QSR International, Victoria, Australia) for Mac. These data included frequency, coverage of the text, and alignment between participants within and across geographic location, respectively. Specifically, these data included frequency across the interviewees, controlling for response length. Taken together, the emergent themes (i.e., as a result of the qualitative analysis of text to identify key themes, Creswell & Poth, 2016; Saldaña, 2015) represented proportions across sites, and these data were important to demonstrate the fidelity across sites, as opposed to reflecting just a singular, dominant respondent during focus groups at any one site.

Next, we coded each interview and survey by location/institution such that we could compare their associated themes. We then refined our list of emergent themes for redundancy and overlap based on the responses and finalized the emergent themes, as described previously (Nelson & Cutucache, 2017). Respondent validation was used to establish credibility and reliability and ensure that students’ perspectives were accurately captured, and multi-researcher read-throughs and interpretations were integrated to minimize bias (Moustakas, 1994).

### Evaluation: program quality assessment

To address the first research question, we used program quality and features as indicators for determining and monitoring fidelity of program replication. Without these metrics, we would not know if the program was actually replicated with significant ownership, or if a fully different program had been created because of “too much” adaptation. Therefore, we utilized existing assessment instruments, relying heavily on the Youth Program Quality Assessment (YPQA) instrument from the David P. Weikart Center for Youth Program Quality to determine quality of programming (David P. Weikart Center for Youth Program Quality, 2020). A researcher from the NE STEM 4U program, certified as an external evaluator for YPQA, completed the assessments. In addition, we used the Harvard PEAR group’s Dimensions of Success tool (PEAR, 2020) to observe and evaluate the undergraduate mentors on their lesson design and implementation, but we did not use this instrument as a proxy for program quality, because each site was able to customize their lesson type. For example, one site included traditional STEM topics (e.g., Biology, Ecology, Geology, Chemistry, Physics, Engineering, Mathematics) and others added emerging and cross-cutting concepts in their activities that include STEM and non-STEM content (e.g., Network Science and Physical Education).

Similarly, we captured the number of youth attendees per instructional experience, an average of 6.9 attendees (range 2–20 attendees) per experience for Fall 2019 sessions, and recognize the danger of too much emphasis on purely quantitative metrics. Attendance can reflect many factors that are out of the control of the program in out-of-school time programs. For example, student illness, early pick up of youth for doctor’s visits, extra homework help, and frequently, competition with sports and extracurricular activities or not can shape attendance numbers. Rather, we implemented observations using established evaluation rubrics (http://www.cypq.org/downloadpqa), ranging from DoS to YPQA to represent the overall fidelity of the program from learning environment through youth voice.

### Replication model: voices from the field for contextualization of implementation

Finally, our institutional partnerships have been vital for the original NE STEM 4U program, we requested that the lead team member from each geographically distinct site (i.e., the original site of origin/initiation, and two franchises) write a summary of their experiences. These voices from the field were implementer observations from those that franchised the original program at their own location. These voices provide contextualization to the goals of the individual sites and regions while also identifying specific challenges and opportunities as a result of the franchise, distinct from the initiation site. We asked the three leaders to describe their vision of the program, why they wanted to replicate the program, and key challenges that they had to overcome to host NE STEM 4U at their location (Table 1). These sources of data all informed the themes that emerged and the answers to the core questions.

**Table 1:** Reflections and perspectives from faculty leads at each site

Site	Faculty Lead Perspectives
Large, urban, metropolitan university (R2)	*“The NE STEM 4U program has enhanced our institution and our community in so many ways. New partnerships and collaborations, both on campus and with our community stakeholders, are directly the result of establishing, strengthening and growing the NE STEM 4U program. Within the university, NE STEM has broken though long-standing silos to engage students, faculty and administrators from multiple units and colleges. It has also been a contributor to growing a new STEM Center administrative structure to increasingly support such programs. Within the community, solid and sustainable collaboration with schools and education advocacy groups has broadened our perspective and our reach. These new relationships built a foundation for new STEM initiatives that have resulted in funded discipline-based research programs addressing a wide range of important questions. As such, the program has enhanced faculty success and contributed to institutional excellence. Most importantly, NE STEM 4U has been fully transformative for our participating students. From the beginning, undergraduate students have taken advantage of the leadership and professional development opportunities of the program and middle school students in our community have responded enthusiastically to the programming. The benefits of NE STEM 4U for undergraduate students and for K-12 students have been documented and disseminated. After six years of developing and refining the program, incoming undergraduate students from many disciplines seek to join because they know about the opportunities for professional development. Some students wish to enhance their leadership skills, some wish to give back to the community, some wish to become better researchers, some wish to become better teachers. Some wish to do it all. We can point to many examples of students who have indeed done it all.”*
Large, research-intensive land-grant institution (RI)	*“Partnering with NE STEM 4U provided vital infrastructure, plans, guidance, training, and a built-in comparison group for our NIH grant. It was vital to have regular communication in order to learn what was assumed by the original site and needed to be explicit for our development and growth. We are starting small (one school the first semester, one school the second semester, two schools the third semester). Trying to create new activities for an emerging science field at the same time as we were learning how to be an NE STEM 4U site was very challenging and exciting. Having our mentors visit the original site to observe and learn, weekly meetings with the original site’s leader, and determining what we would adopt (e.g., formats, hiring expectations) and what we would not adopt (e.g., we did not start as a registered student organization). The challenge that we still have is how to find a permanent home for NE STEM 4U. We have 3.5 more years of funding and currently the program is in a Sociology Department, not a physical science department. We have learned what should be part of fundamental mentor training and what should be explicit in activity plans. Finally, our original focus was on developing middle school youth science identities and interest in health careers where the emphasis for the original program was on mentor professional development and retention in STEM. Therefore, we had to learn that we needed to also focus on mentor professional development and mentor community development. Having the ongoing coaching, support, and guidance as we grow our NE STEM 4U franchise has been vital to our continuation. In our third semester, we are at the point where we can mostly have the program run with less intense faculty/staff oversight and can also focus on developing more activities. Having our evaluators learn the Dimensions of Success (DOS) evaluation tool to provide feedback on emerging activities and the YPQA evaluation of the mentors gave us the necessary information to make ongoing improvements. We have also learned what to look for in hiring mentors and how to incorporate the mentor handbook and weekly reflection tools to support effective mentor development.”*
Small, predominantly teaching, rural institution (PUI)	*“The vision of the NE STEM 4U program at our university was to provide undergraduate students the opportunity to enhance their knowledge base of STEM, develop leadership, oral and written communication skills, and provide the opportunity to educate children (elementary & secondary) using their pedagogical background in their undergraduate degree. Our program was unique for we had mentors teach the NE STEM 4U science lesson and also included physical activities to assist the children in gaining a better understanding of STEM concepts taught for the day. We wanted to replicate the program because it utilized experiential learning and created a bridge to teach cross-curricular topics in a fun and creative way. We felt it had the potential to increase interest in STEM fields for both children and undergraduate mentors. The original franchise program already had a strong framework of lesson plans, so we were able to smoothly incorporate STEM based physical activities. The main challenges our program encountered were related to us being a new program. We learned the mentors needed to build relationships and trust with NE STEM 4U faculty, and with the children prior to having full buy-in of the program. The first semester our mentors dealt with behavior and classroom management issues which then transferred into difficulty in completing the teaching of the lessons. We believe the undergraduates were not adequately prepared, and made adjustments moving into the second semester of activities. The new mentors began with team building activities with NE STEM 4U faculty, and then utilized team building activities related to STEM concepts during the initial session with the children. These changes have positively addressed our initial challenges at the start of our NE STEM 4U Franchise.”*

The description of ‘undergraduate’ by the responders is specifying post-secondary students at the university level

## Results

### Research question 1. Is the program replicable?

Stated another way, can we take the initial, core program and ‘franchise’ or replicate it within a different context, with other program leads, and in an environment with a mission distinct from that of the initial site? To address the first research question, we utilized *quality* of out-of-school time programming and presence of core elements as a proxy for the ability to replicate the program. The David P. Weikart Center for Youth Program Quality instrument provided consistency and assessor calibration so that the main variable was original for franchise location. The YPQA results (Table 2) demonstrate general replication of program quality in a “franchise” location. Although somewhat lower, the average scores at the replication sites (R1 and PUI) *Supportive Environment* (4.51) and *Interaction* (3.13) compared to the original large, urban, metropolitan university (R2) were mostly similar (4.72 and 3.25, respectively). The replication sites had lower scores for *Engagement* (2.21) compared to the origin site (2.99), indicating a need for potential improvement. Even though implementation happened over multiple sites and years the quality of program delivery was mostly similar. The franchise sites maintained core program components (namely: (i) intentional programming, (ii) staff quality, (iii) effective partnerships, and (iv) program evaluation and continuous improvement). Of note, the ‘program evaluation’ component is characterized using the same *assessment instruments* as opposed to the same evaluator—i.e., using the YPQA and DoS instruments. Ultimately, the evidence indicates that it is possible to replicate the program and maintain quality. We explore the core components more below.

**Table 2:** YPQA internal evaluations of the NE STEM 4U program

	Large, Urban, Metropolitan University (*R*2) (*n* = 16)		Expansion sites (*R*1 and PUI) (*n* = 4)	
	Average	Range	Average	Range
**Supportive environment**	**4.72**	**4.53–5**	**4.51**	**4.03 – 4.73**
Warm welcome	4.67	4.33–5	4.50	4.33–5
Session flow	5	5	5	5
Active engagement	4.59	4.5–5	4.25	3–5
Skill building	4.97	4.5–5	4.63	3.5–5
Encouragement	4.42	3.67–5	4.17	3.67–4.33
Reframing conflict	3.5	3.5	N/A	N/A
**Interaction**	**3.25**	**2.29–4.04**	**3.13**	**2.45–3.58**
Belonging	3.66	2.5–4.5	3.38	2.5–4
Collaboration	3.67	1–4.33	4.33	3.67–5
Leadership	3	1.67–4.33	2.33	1–3
Adult partners	2.69	2–4	2.5	2–3
**Engagement**	**2.99**	**2–3.83**	**2.21**	**2.17–2.67**
Planning	2.44	1–4	1.5	1–3
Choice	2.38	1–4	1.5	1–2
Reflection	4.19	2–5	3.63	3–4.5

Internal evaluations conducted by a certified evaluator on the NE STEM 4U program using the David P. Weikart Youth Program Quality Assessment tool. The evaluations were conducted between August 2019 and February 2020. Due to IRB limitations and video quality, only a limited number of evaluations were allowed on expansion sites. Bolded text indicates key differences between the *R*2/initial site and the expansion sites (*R*1 and PUI institutions)

### Research question 2. What core components are necessary for program fidelity?

We now review some quantitative and qualitative components related to fidelity.

#### Quantitative indicators

We interpreted that the sheer length of time a partner site was still running the program (i.e., at least 1.5 years) indicated successful replication. Similarly, the recruitment of mentors (and new mentors, as others graduated/left the program, *n* > 150 to-date), indicated sufficient demand for the program, as well as demonstrated buy-in from the post-secondary students. Because the original NE STEM 4U program was primarily about the professional development of post-secondary STEM majors, the ability to keep recruiting mentors is an important sign of fidelity.

Each programming location (i.e., the partnering out-of-school sites) maintains an attendance sheet that provides information on how many youth members participate in clubs. The YPQA evaluation observation days each had at least 6 participants, and some had as many as 20 youth participating in NE STEM 4U on any given day. Our suggested cap on attendees is 15, thus whenever we met or exceeded this number, we interpreted this as demand for the programming. Several factors (e.g., weather, competing programs, team sports, school site-specific rules) could influence attendance so that the measure is suggestive but not definitive.

### Replicability of expansion sites: ensuring fidelity of a ‘Franchise’

To be a “franchise” or replication site with co-ownership, a location had to have post-secondary students go through training similar to the original location (‘staff quality’), use activity plans provided by or modeled on the original program (‘intentional programming’), allow for evaluation (‘program evaluation and continuous improvement’), focus on community building and professional development of mentors, and develop and maintain positive relationships with Community Learning Center staff (‘effective partnerships’) (Fig. 2).

Slight variation in approach, however, was approved by the originating team. For example, one franchise had weekly clubs, where another hosts twice-weekly clubs. One site includes 4th and 5th grades and others only middle school grades. One NE STEM 4U program operated as a student run organization (temporarily) and another was funded by and led by a federal grant with heavier faculty and staff oversight. Given these baseline characteristics, the question that remained was this: can the franchises reach the level of quality or respond to feedback to achieve the level of quality of the original site? The YPQA evaluations suggest that the franchise teams could use the key elements (i.e., involvement of post-secondary students as mentors, out-of-school time inquiry based STEM activities with youth, and mentorship from leaders of the program) of NE STEM 4U to create engaging and enriching programs at each location.

**Fig. 1:**
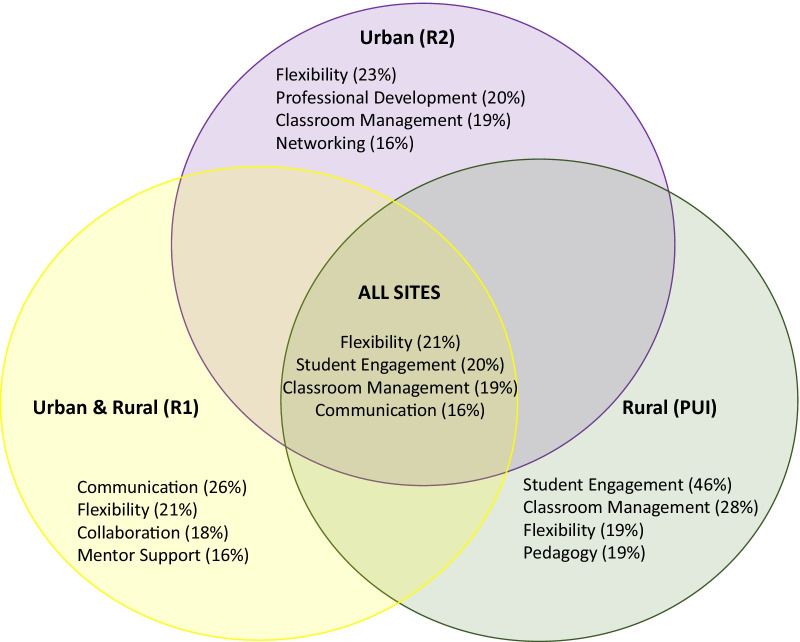
Emergent Themes from the NE STEM 4U program. Identification of the emergent themes (based on percent coverage in parentheses), from coded focus groups and survey responses across all sites. The unifying, consistent emergent themes across franchise sites are described (All Sites), as well as uniquely across each franchise site (Site of Origin, a large, urban, metropolitan university (*R*2)), and both expansion sites (Urban and rural-mixed (RI), and a Small, Rural University (PUI)). Specific themes were unified across all sites

The data from post-secondary student mentor focus groups and reflection surveys (Fig. 1, Table 3) suggest similar “takeaways” from the experience at each site. Moreover, the program can be successfully replicated in both rural and urban settings, and at institutions of varying size with differential prioritization of research and teaching. Therefore, the answer to the first research question (RQ1) is yes, expansion sites can replicate core elements of the NE STEM 4U program. In addition, the answer to the second research question (RQ2), in the process of exploring if the expansion sites were faithful to the original program, the leaders of the original and expansion sites realized that “fidelity” means more than simply adopting the exact practices of the original program. Rather, as mentioned previously, the essential components were generally: mentors delivering the content, the activities/lesson plans, and mentorship surrounding the programming from leaders (e.g., the youth voice, culturally relevant pedagogy, active learning strategies). Finally, the answer to the third research question (RQ3) is that NE STEM 4U can be effective in various settings (e.g., rural, urban, and geographically distinct regions) with minimal adaptation.

**Table 3:** Emergent themes observed in post-secondary student/undergraduate mentor quotes

Emergent theme	Undergraduate mentor quotes
Flexibility	*“I think I enjoy changing the lessons and kind of just forming them to our needs.”* *“I think allowing the mentors to have a lot of flexibility and freedom ensures that the program stays very relevant and customized to each classroom.”* *“I think that kind of flexibility and reliance on the opinions of those who actually executed the lessons was a definite good, strong point.”* *“I think flexibility is really critical, because some of the time the lessons aren’t as engaging for students…. So I think being flexible, while you’re in the classroom and while you’re practicing and working with your other mentors is really important.”*
Student Engagement	*“The students had a lot of fun being able to make their own candy blood vessels and being able to see it and play with it.”* *“I would say the most important part [of NE STEM 4U] is trying to get as many kids involved and interested as possible in the activities and make sure they can understand and have fun with the experiments.”* *“Having the enjoyable activity is a really important part so they get the interest of the science and then after having this enjoyable activity they should take something from it.”* *“When [students] are interested in the experiment it is really neat.”*
Classroom Management	*“I feel that not having knowledge in behavior and classroom management techniques and how to teach would make it challenging to maintain an efficient learning environment.”* *“…developing my skills, how to communicate and deal with kids, this is the big thing.”* *“[In professional development training] we talked about managing not necessarily conflict but just like managing a classroom.”* *“I think that [professional development opportunities] that we have where we are teaching things like classroom management and useful skills that are supper important… make people more effective in their mission.”*
Communication	*“I think the communication has worked well and that we are getting a lot of students involved.”* *“I think open communication has worked really well. [Liaison between sites] has been really great about communicating with us and helping us throughout everything.”* *“Developing my skills, how to communicate and deal with kids is a big thing.”* *“Also with the staff that’s helping us [communication] is really critical. Just like build those relationships, it helps us understand and it helps them understand where we are coming from when we are trying to change things, or we are confused, or just stuck.”*
Professional Development	*“I’ve gained a lot of leadership skills where I am more comfortable leading a classroom of elementary and middle schoolers.”* *“In general I’ve had the opportunity to work more on the organizational side of things and working as part of a team just things like public speaking, planning events, professionalism, making contacts in the community, there’s definitely a lot of that as well.”* *“Just gaining that professionalism and being more comfortable talking in front of a group of people or just seeing what you need to do, playing it out, and executing it has been very beneficial.”* *“I think accountability is really important, mainly peer to peer, or peer to officer.”*
Networking	*“I interacted with some of the other teams that are starting up programs.”* *“I think it was beneficial prior to the start of the semester when we had all of the different groups and different mentors [together], everyone came and we kind of all met and talked about different strategies at different schools do things and that was helpful.”* *“I think interacting with the other mentors definitely makes you aware of how many things… I’ve picked up along the way.”* *“I would say connections, like the network of schools you are in and you know the teachers and other mentors that are there and there’s a lot of different afterschool programs that are working together so there’s a lot of connections and relationships that can be built there to help you in whatever you’re going to do later.”*
Collaboration	*“I do not have a lot of knowledge of the body and muscles so it was difficult to teach but it went well with [peer mentor]’s help* *“It allows college students to collaborate with people from other majors and to achieve the goal of teaching kids in other sites.”* *“One of my favorite lessons from last year was from one of the new sites, a lesson that they had developed and shared with us, so I think the opportunity to get more resources and more ideas to implement our lessons is definitely a bonus.”* *“It’s nice to have a partner to fall back on too, if you don’t necessarily know the best way to handle it you have someone who can step in too, you’ve got two of you there to help situate it.”*
Mentor Support	*“Our superiors have been very helpful just bouncing ideas off of each other and keeping that open line of communication.”* *“We’ve sent mentors in fully equipped with a lesson plan that is comprehensive.”* *“I wish our location had their own personal training day that was specific to our needs.”* *“We’ve set our mentors up with training and peer and faculty support.”*
Pedagogy	*“The lesson should be kept but the way of teaching it needs to be changed. Maybe they can make one as a whole group and working together to try and complete the tasks.”* *“I would keep this lesson but I would reteach it so it is more of group work or make it more interactive.”* *“For my major I’ve gained like building a lesson plan and changing that to help the students, I think that’s very valuable.”* *“The prep out of the classroom requirements is significant but necessary in making that hour in the classroom actually count.”*

Example quotes from post-secondary/undergraduate mentors referencing all emergent themes from overall franchise sites and each individual franchise site

#### Qualitative emergent themes

The perspectives from post-secondary student participants or ‘mentors’ are detailed in Fig. 1, with specific quotations highlighted in Table 3. Importantly, we examined these emergent themes by each site, as well as in combination (*n* = 16 focus group participants, *n* = 12 reflection surveys). These data indicated that *Flexibility* (21.22%)*, Student Engagement (i.e., Youth)* (19.53%)*, Classroom Management (i.e., also pertaining to youth)* (19.31%)*, and Communication* (15.71%) were the themes most referenced by the post-secondary student mentors in the NE STEM 4U program, regardless of site (Fig. 1, Table 3).

Participants from the founding institution identified the following key themes (*n* = 12 focus group participants): *Flexibility* (23.18%), *Professional Development* (20.23%), *Classroom Management* (18.75%), and *Networking* (16.26%). Importantly, the percent coverage of specific software nodes to articulate emergent themes reflects consistent responses across participants—not that only four of the twelve participants mentioned ‘flexibility’, for instance. Participants from the RI institution replication site generated the following themes (*n* = 2 focus group participants): *Communication* (25.57%), *Flexibility* (21.01%), *Collaboration* (18.29%), and *Mentor Support* (15.81%). Importantly, at this franchise site, given its early stage, there were only four mentors; thus, this focus group represents half of the mentors at this site (*n* = 2). The limitation of responses here are simply because of the short duration of replication at this site thus far. Participants from the PUI institution replication site described experiences that we codified as (*n* = 2 focus group participants, *n* = 12 reflection survey participants): *Student Engagement* (i.e., engagement of the youth in the program) (46.21%, *Classroom Management* (28.34%), *Flexibility* (19.46%), and *Pedagogy* (18.70%).

The faculty leaders at each location identified more structural, training, and management challenges that are less apparent than those identified by the post-secondary student mentors (see Table 2). One leader described challenges in training mentors, realizing that in addition to mastering the content and implementation of the activities, it is vital to provide preparation, ongoing professional development, and community building for the mentors. Furthermore, leaders must ensure program quality while also developing activities in new areas. One particular challenge for faculty leaders was determining how prescriptive written activity plans should be, given what is discussed in mentor training. Another challenge was related to the dual goals of the program; the differing emphasis on developing and retaining post-secondary mentors on the one hand and attracting middle school aged youth to STEM careers on the other. Faculty leaders included their own reflections on why they were interested in participating in the project to begin with and their observations on other programmatic implementation issues to-date.

### Research question 3. Is there a dependency on a particular setting/participant type (be that a more rural or urban setting)?

**Fig. 2:**
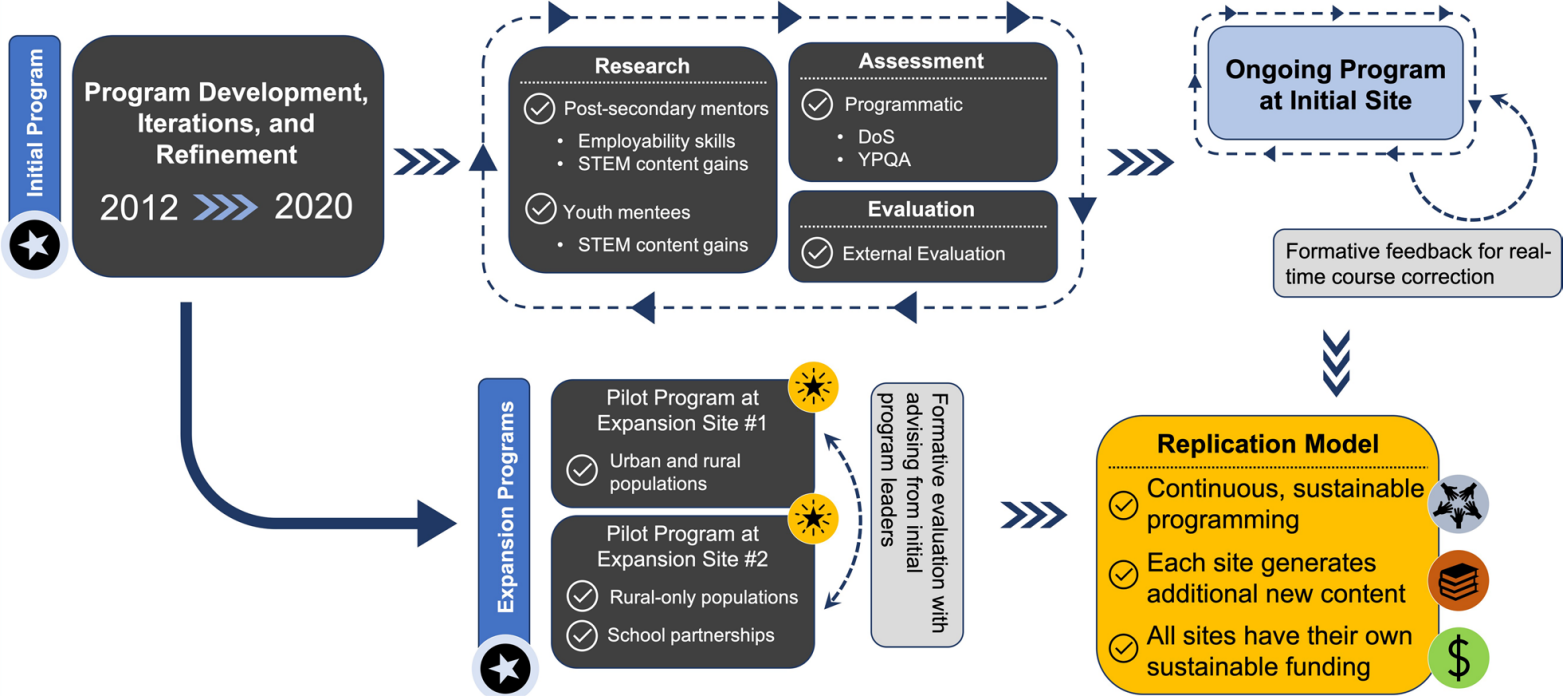
Illustration of the Iterative Process from Initial Program through Expansion Sites. Illustration of the iterative process from initial program through expansion sites (the so-called ‘franchise sites’) and the feedback loops accompanying each. The filled stars at the expansion program sites indicate that these were both led by non-STEM faculty, further emphasizing the translatability of a STEM program via this replicable model, regardless of the prior degree and/or training of the program lead. Post-secondary mentors refer to those carrying out the programming for youth within the program (‘mentors’) and the ‘youth mentees’ refer to participants within the program. *DoS* Dimensions of Success, *YPQA* Youth Program Quality Assessment

Given that the founding site of this program was within a large, urban, metropolitan area, and took place at a university embedded within the city and whose mission emphasizes community engagement, we aimed to determine if there was a need for and an impact of the NE STEM 4U program in areas with a different context and mission. Thus, we investigated two additional sites of replication, which were in divergent geographic and demographically distinct regions, all with partners that did not emphasize ‘community engagement’ as part of their core mission (Fig. 2).

The two expansion sites have now been operational for at least 2 years (at the time of preparing this manuscript), have their own funding source (unique of the initiation site), and have their own evaluation and programming, including new content foci and development (Fig. 2), representing significant co-ownership that is often necessary to help a franchise to grow with the support and advice to ultimately succeed locally. These data indicate that the NE STEM 4U program is warranted in a variety of settings, can be replicated to be independent ‘franchise’ locations with success, and is sustainable with independence from initial site. The importance of this finding cannot be overstated—this indicates that the programming is independent of participant type, geographic location, initial funding/seed funding source, and specific discipline within STEM. To achieve this level of replication, it was necessary to have clear guidelines and ongoing communication between the originating/initiation site and franchise sites. Ongoing communication and some shared professional development opportunities among mentors provided continuous improvement through feedback loops and evaluation. For example, the *R*2 site had their mentors complete reflections after each club session and reviewed the information with the initiating team to make corrections if necessary.

### Synthesis of the research on the model of replication

Through our assessment of NE STEM 4U (including post-secondary mentor reflections, program quality via YPQA, and faculty leadership reflections) we have identified four *core elements* of franchise success: (1) Intentional Programming, (2) Staff Quality, (3) Effective Partnerships, and (4) Program Evaluation and Improvement. Specifically, program success depends on the fidelity of specific aspects of these four elements, while other aspects are more flexible depending on site limitations and preferences. In addition, we have discovered that evaluation and improvement is particularly critical for effective lesson plan design, as this real-time feedback helps novice mentors to facilitate a positive, active learning environment, and thereby maintain quality, youth engagement, and youth voice (as supported by post-secondary student mentor reflections, see Fig. 1 and Table 3). Consequently, we have prepared a publicly available link to lessons for utilization in other programs (NE STEM 4U lessons: https://www.unomaha.edu/academic-affairs/ne-stem-for-u/ne-stem-4u-activities/lessons.php). Moreover, the process of determining if a franchise is maintaining fidelity assisted the initiation team with determining what is ‘essential’ and what is ‘flexible’ within the programmatic replication.

The major takeaways based on our observations herein, include the need for “win-win-win” scenarios for franchisees with the founding program and with all NE STEM 4U programs with partners (e.g., Community Learning Centers and schools). These scenarios are predicated upon an ongoing mutual respect with continuous communication. Namely, in the NE STEM 4U program, we are creatively filling multiple needs: (1) offering professional development for post-secondary students to include teaching, research, and mentorship, (2) providing K-8 participants (i.e., grade school age children) afterschool supervision, progressing their learning of content knowledge (Nelson & Cutucache, 2017), and helping to foster an improved STEM interest in hopes of building a STEM identity, and (3) working to add commonality in metrics to evaluate and report on out-of-school time activities to add to the base literature, but also to allow for further longitudinal meta-analyses that help to determine critical types of supports (and at what point(s) in a student’s life) during the development of new scientists. For the franchise sites, the opportunity to take an existing platform (i.e., the NE STEM 4U program) and replicate it within their own site, gave them the opportunity to scale-up on specific research questions immediately, thus aiding in obtaining extramural funding and expediting their core goals due to simply a ‘replicable’ program with established track record. There would certainly seem to be advantages with building upon a successful program, as compared with trying to create something from scratch, or with low fidelity, to ultimately generate more pitfalls than research outcomes.

## Discussion

Within this study, we sought to examine the replication or ‘franchise’ of the NE STEM 4U program to various sites with diverse characteristics. We examined the program quality (using YPQA and attendance as a proxy), post-secondary mentor participants’ key takeaways, and lead faculty perspectives and reflections. Overall, we aimed to determine, via implementation science, NPT framework with dissemination, insights about the program at the original location and at the franchise locations from the perspectives of the post-secondary/undergraduate student mentors.

### Emergent themes demonstrate core elements for replication

Examining emergent themes from post-secondary student participants at the different sites—keeping in mind the diversity of the participants, their backgrounds, and their surroundings—we observed the following. The large, urban, metropolitan institute participants, who were part of the original program and, therefore, the longest existing program, highlighted program elements, such as *Flexibility* (23.18%), *Professional Development* (20.23%), *Classroom Management* (18.75%), and *Networking* (16.26%). These data suggest that the participants have a clear understanding of the support that the NE STEM 4U program provides for professional development. For example, they see the benefits of learning to communicate with others, classroom management, and networking. For the metropolitan cohort, “peer networking” (10.98%) is important (the 4th theme) and suggests the community of mentors provides additional value. Gaining insights from the perceptions of the mentors adds to other data on the metropolitan mentors about the benefits of participating in NE STEM 4U for outcomes, such as critical thinking gains and retention in major and to graduation. There is a strong track record of degree completion (97%) and retention in a STEM career post-graduation (96%; Nelson et al., 2018), as compared with the national U.S. rates of 40–60% (HERI, 2010). Therefore, participating in NE STEM 4U may benefit, and certainly does not hurt, graduation and STEM major retention.

The participants at the first expansion site, a research-intensive, flagship institution (with a much larger student population but smaller metropolitan size) identified *Communication* (25.57%), *Flexibility* (21.01%), *Collaboration* (18.29%), and *Mentor Support* (15.81%), as integral to NE STEM 4U. The research institution NE STEM 4U franchise just completed 2 years and had no mentors carry over from the first semester to the second semester, due to the cohort of student mentors being all seniors and thus graduated at the completion of that year. Through conversations with the founding NE STEM 4U team, the franchise realized that to build a professional community of mentors that will carry over semester to semester, there is a need to start with students earlier in their career (e.g., sophomores) and to focus on professional development and community building among the mentors. Therefore, from the second implementation onward, sophomore-level students were included. However, of the participants, the mentors (who had participated for one semester) valued collaboration and direct support via training. We utilize the ‘train the trainer model’, and collaborative professional development workshops via Zoom (video conference) and FlipGrid™ in an effort to build community and collaboration across sites. These are publicly available non-negotiable of the dissemination of the intervention. Similarly, faculty advisors (and in many cases, a graduate assistant or program coordinator, also known as ‘site leaders’) serve as daily points of contact for post-secondary mentors, further giving them a community in which to belong and be confident in trying new things, such as outreach programs.

The predominantly teaching-focused, research-active institutional partner in a smaller town (i.e., 35,000 people in the town) had student participants describe *Student Engagement* (i.e., Youth) (46.21%, *Classroom Management* (28.34%), *Flexibility* (19.46%), and *Pedagogy* (18.71%). It is interesting that given the teaching charge of the institution, the identified core themes are consistent with the mission. Finally, despite a unique geographical location (the most rural of our sites), participants identified the same themes (flexibility and classroom management) as participants at the urban institutions, and suggesting minor influences from the institutional norms.

### Fidelity in core elements

None of the data collected and analyzed contradicted the core elements of quality program replication identified by other programs seeking to grow (e.g., the Afterschool Alliance, 2014). The core elements necessary to create a successful ‘franchise’ are: (1) Intentional Programming, (2) Staff Quality, (3) Effective Partnerships, and (4) Program Evaluation and Improvement. Comparing mentor and faculty leader experiences and perceptions at the three sites (initiation site, and two franchise sites) provides evidence that the core elements emphasized by national leaders, such as the Afterschool Alliance for high-quality, sustainable programming apply to the NE STEM 4U program as well. Therefore, these data emphasize the translatability of the core elements of programs, such as the NE STEM 4U, across multiple sites for post-secondary/undergraduate student engagement, professional development, and preparation as scientists. In addition, the program is flexible enough to incorporate a franchise developing activities in an emerging area of science as well as learning and implementing existing activities. Importantly, our data also indicate that NE STEM 4U can be translated to other franchises flexibly if there is ongoing communication with the originating team site (see faculty leader reflections—Table 1, and post-secondary student mentor reflections—Fig. 1 and Table 3), integrating each individual franchise’s goals while maintaining the core elements of the program and the quality and integrity that is integral to the success of NE STEM 4U.

### Limitations

The key limitations of this work are the relatively short duration of the program franchise sites; specifically, while the initial site has been operational for 8 years, the two expansion sites have been in operation for only 2 years each, which meant that fewer mentors were present at these sites due to the short duration of operation thus far (Fig. 2). In addition, all sites have followed a process similar to that of a commercial franchise, wherein the ‘branding’ and core elements came from the initial site and the initial site provided coaching as needed to each expansion site. As the model replicates on a broader scale, it will become clear how/if the coaching as needed is either simply helpful or if it is a crucial part of the model. Finally, this model has only been replicated within the United States, within a singular state so far and has not been implemented in other regions of the country or world. However, the anchor author and founder (Cutucache), has received a Fulbright Fellowship to carry out this specific work within Finland and has a collaboration with an Australian team also underway, further emphasizing the international translation and relevancy of the program, and mobilizing the opportunity to determine programmatic integrity elements for an international context. Therefore, the NE STEM 4U program has the potential for broader implementation in years to come.

## Conclusions

In conclusion, we provided the core elements for fidelity of the replication of the NE STEM 4U program, as well as emergent themes from participants, from its initiation site to that of its expansion sites. We report four core elements that ensure the fidelity of our program are: (1) Intentional Programming, (2) Staff Quality, (3) Effective Partnerships, and (4) Program Evaluation and Improvement. Moreover, we identify emergent themes across all sites to include: *Flexibility, Student Engagement, Classroom Management,* and *Communication.* These findings are in direct support of the constructivist theoretical framework, and demonstrate the value of the program for STEM undergraduates interested in entering STEM teaching professions. This highlights the potential impact that this program may have for greater retention in the pipeline to address the shortage of STEM teachers. Moreover, through connecting student mentors with professionals across STEM disciplines, our program helps post-secondary/undergraduate students see that there are more jobs available than simply those routinely touted (i.e., physician). We connect mentors with in-service teachers, informal educators, active researchers in science education, policy makers, administrators within research and development, intellectual property professionals, and data analytics scientists, hopefully also opening the door to the array of fields available in STEM disciplines.

Finally, and perhaps even more importantly we demonstrate through normalization process theory that this longstanding program, with demonstrable impact and quality, can be replicated or ‘franchised’, thus expanding impact. Finally, we provide rationale for the replication of an existing program as a way to expedite research at various locations, without the delay of a re-design. We have demonstrated herein that the scale-up approach for this program can be completed independent of participant type (rural or urban environment) and in geographically distinct regions, thus enhancing the potential for replicability internationally. Consequently, our findings are important and relevant to the field of STEM outreach to aid in developing and enhancing the STEM pipeline.

### Future directions

Subsequent work will investigate, based on these identified emergent themes, if we can also follow the cognitive development of students via Chickering’s Vectors, as part of Student Development Theory. We aim to track post-secondary students over time and identify if NE STEM 4U aids in providing a ‘series of stops’ on the student development theory railway. In this, we will investigate if aspects of NE STEM 4U underlie the development of post-secondary students from both a cognitive development and workforce preparation perspective. Similarly, while we have previously reported the impact on participants 3 year post-experience (i.e., 3 years after graduation/3 years into either graduate school or the workforce, Nelson & Cutucache, 2017), we also aim to determine if the methods of training need to be adjusted for incoming students. With the addition of some social science health related activities, we also need to assess if students with less training and lower measures of conventional STEM identity can succeed as mentors.

Finally, we would be remiss if we did not point out the key partnerships that are common to these programs supporting initial fidelity conversations and that may have been contextually helpful in replication. Several partnerships are similar across the three locations: school systems, universities, non-profits, and agencies. All of the institutions also prioritize cultural relevancy through targeted training. Successful programs are integrated with webs of connections to partners with shared missions, and NE STEM 4U at all three locations is no exception. Future research might explore if particular partnerships and relationships are crucial to success. For example, an early attempt to create a franchise at the more rural teaching-focused institution did not persist without a dedicated university champion. Not surprising and worth emphasizing, expanding to additional locations requires a carefully crafted strategy of focused care and attention to maintaining the core features of a successful program and adaptation to local needs. However, it also has advantages in improving the whole program. Direct attention to data collection and evaluation, not only on the quality of the program but also fidelity, and when it is necessary to adapt, is vital to growth both in the lead site and elsewhere. Exact word for word translations do not always capture the essence of a phrase, and similarly, keeping focused on the essence of the program and determining what should and should not be incorporated in a new setting is valuable for dissemination and growth of quality STEM programs.

## Supplementary Information

**Additional file 1. Table S1.** Focus Group Survey Questions.

## Data Availability

The data sets used and/or analyzed during the current study are available from the corresponding author on reasonable request.

## Abbreviations

NE STEM 4U: Nebraska Science, Technology, Engineering, and Mathematics (STEM) 4U
YPQA: Youth Program Quality Assessment
DOS: Dimensions of Success
K-8: Kindergarten through 8th grades
