# Supplementary material for: Replicating or franchising a STEM afterschool program model: core elements of programmatic integrity
Source: Int J STEM Educ. 2022 Jan 28;9(1):10. doi: 10.1186/s40594-021-00320-0 (PMC8795932; doi:10.1186/s40594-021-00320-0)
Supplement: Supplementary file 1 — Additional file 1. Table S1. Focus Group Survey Questions. [file 40594_2021_320_MOESM1_ESM.docx]

**Additional file 1: Table S1**

*Focus Group Survey Questions*

| 1. What do you think is the most important/valuable part of the NE STEM program?    1. To you, as a mentor    2. In general (if previous answers aren’t descriptive) |
| --- |
| 1. (define first, what is the franchise specifically, so they know context) |
| 1. What has been your involvement with the franchise process of NE STEM, how much do you know about the process? (To other sites/from other sites—depends on mentor group). |
| 1. What do you like most about the franchise model of NE STEM? |
| 1. What do you think has worked well in the franchise process of NE STEM? |
| 1. Has anything surprised you in the franchise process of NE STEM? |
| 1. What have you, as a mentor, gained from NE STEM, and the process of expanding NE STEM to other sites? |
| 1. What do you think is most critical to retain in NE STEM at other sites? |
| 1. What do you think can be flexible when expanding NE STEM to other sites? |
| 1. What are challenges associated with NE STEM?    1. Challenges with expanding to other sites? |
| 1. How can those challenges be addressed?    1. From the mentor perspective    2. From the franchise programming perspective |
| 1. What do you wish had been done differently in the NE STEM franchise process? How could the process of expanding have been more helpful to you as a mentor? |

*Note.* Open-ended focus group and survey questions. Questions were asked of the post-secondary (undergraduate) participants across sites to determine emergent themes.
